# On the Treatment of Tuberculous Disease of the Hip-Joint

**Published:** 1896-10-03

**Authors:** J. Lacy Firth

**Affiliations:** Assistant Surgeon Bristol General Hospital.


### Medical Progress and Hospital Clinics.

[*The Editor will be glad to receive offers of co-operation and contributions from members of the profession. All letters should be addressed to* The Editor, at the Office, 28 & 29, Southampton Street, Strand, London, W.C.]

### ON THE TREATMENT OF TUBERCULOUS DISEASE OF THE HIP-JOINT.

By J. Lacy Firth, M.D., M.S. Lond., F.R.C.S., Assistant Surgeon Bristol General Hospital.

Most surgeons will agree with the statement that in the early stages of strumous coxalgia it is wrong to at once attack the disease by operation. In this, as in other parts, the ravages of the tubercle bacilli may be, in many cases checked without bloodshed. The disease will in a large proportion of cases be cured, and recovery ensue with a limb but slightly impaired in function, if from the outset rest be maintained, by the application of a weight extension or a splint, or both, and if appropriate constitutional treatment be at the same time adopted.

But all surgeons are not agreed upon the treatment which should be applied when caseation and suppuration are detected. Some maintain that in this stage excision of the joint is called for, others that it is not; but all recommend the evacuation of the abscess contents with aseptic precautions. The treatment by simple incisions and scraping, and that by excision of the diseased bone, have both been tried upon an extensive scale. It is remarkable, therefore, that there is still in existence such a diversity of opinion as to the relative merits of the two methods.

Some few surgeons can perhaps still be found who would advocate the performance of excision in an extremely early stage of the disease, before caseation can be detected. These surgeons treat the diseased area much as they would a malignant growth. Their disciples will be few. The cases they operate upon would often recover without operation, leaving much better limbs, and the hopes which they entertain of eradicating the disease by a limited operation, which will very slightly impair the functions of the joint, are usually doomed to disappointment. The operations in this early stage must be fairly extensive to completely expose and remove all the affected synovial membrane and bone, and prevent recurrence, and the joint functions will be impaired to a corresponding degree. These surgeons sail, as it were, between Scylla and Charybdis, being so likely to needlessly impair the joint, or fail to cure the disease. I say needlessly impair the joint, because so many of the cases would recover with a better limb without operation. There is, however, one class of early cases which may be cured by operation without incurring the risks just mentioned. They are the cases in which the tubercular disease is still focal in the neck of the femur, usually in newly-formed bone of the diaphysis, close to the epiphysial cartilage. The operation which has been recommended for these cases is that of tunnelling along the neck of the femur from the trochanter with a half-inch trephine, until the focus of disease is reached and removed. The joint is not opened, and its functions are, therefore, left perfect. Mr. Barker (Treves' "System of Surgery," p. 1,095) speaks favourably of this, and he describes the signs and symptoms by which this variety of coxalgia may sometimes be recognised. The symptoms are a sense of uneasiness in the joint, rather than actual pain, leading the patient to limp, and when the joint is examined it is found that fairly free movement is permissible, but that forcible passive movement elicits pain, probably because the head of the femur is then forcibly pressed upon by the acetabulum. For the same reason jars upon the knee or trochanter, forcing the head of the bone into the acetabulum, cause pain. On the other hand, if the synovial membrane is involved, all passive movement is resisted, and the joint fixed, by which friction of the inflamed surfaces is prevented. Also the characteristic position of acute synovitis is assumed by the limb. These signs, however, do not serve to exclude the acetabular variety of the disease.

Mr. Pickering Pick (*Clinical Journal*, July, 1896), does not recommend tunnelling the neck of the femur. He waits for evidence of caseation, and then if the disease is limited to the neck, he gouges out the affected area of bone from the front, exposing it by Parker's anterior incision. He points out that in such cases the joint itself may escape and the pus form in the extra-articular tissues, because adhesions of the layers of synovial membrane which cover the neck have formed, through which the pus burrows. He has found in a few cases a small area of bare bone in the neck of the femur in front, where the disease has come to the surface, and there he applies the gouge.

Mr. Marsh considers a large operation in early cases totally uncalled for. He says (*Disease of Joints*, p. 325): "I am convinced that in such instances the mortality is not more than at most 5 per cent., and that, in a large proportion of the cases (70 per cent., I should say), recovery with but slight lameness and slight loss of movement takes place."

In the next stage of the disease, when caseation and suppuration are established, some form of operative treatment is now always adopted. It must be remembered that caseation does not always imply the existence of destructive arthritis. Whether the disease has begun primarily in the head or neck of the femur, or in the synovial membrane, or in the acetabulum, a localised abscess may form which is extra-articular. This is, however, very exceptional; and nearly always an abscess formation, in the second stage of hip disease, means that all the component parts of the joint are affected, and that if the object of the surgeon be to eradicate the disease by operation, he must excise the joint. If it were found, after the abscess cavity had been emptied of its contents and thoroughly scraped and flushed, that there was no grating in the joint with movement, and no distension of its capsule from the presence of pus, the proper treatment would be to close the wound, with or without drainage, and endeavour to secure obliteration of the cavity by primary union of its walls. Mr. Marsh has ably argued in favour of treating the majority of the cases of this group by incisions and scrapings of the kind just described. He says (*op. cit.*, p. 325) that with this treatment "deformity may be removed by weight extension, and recovery will be secured with very little shortening (less than an inch), often with movement that is but slightly impaired, and with very slight lameness."

Mr. Pickering Pick (*op. cit.*) is guided by the condition of the parts discovered at the time of operation. If he finds the head of the bone, though denuded of cartilage, hard and resistant to penetration by the probe, he contents himself with scraping the head of the femur (and adjacent parts) as well as it can be done without turning it out of the acetabulum. In some cases he has succeeded in curing the disease in this way after draining for some weeks. He admits, however, that half the cases fail, and excision has subsequently to be performed. The cases which succeed have a better limb than excision yields. He also excises in all cases —and they constitute the great majority—when the head of the femur is found to be soft from caries and easily penetrated by the probe. Caries of the head is of such frequent occurrence because the disease usually begins near the cartilage of the epiphysis.

Mr. A. E. Barker is another advocate of excision in this group of cases. He says (*op. cit.*): "When it becomes evident that liquefaction of the soft tissues of the joint is taking place, and that the bones are becoming destroyed, I feel justified in assuming that the ultimate results of operation for the removal of the diseased tissue will be at least as good as those arrived at by the expectant mode of treatment, and that they will be in one respect very much better—*i.e.*, in saving much time to the patient." Mr. Barker holds that in these cases more bone will be destroyed by the disease under expectant treatment than by the operation.

It is obvious, then, that the question of excision is still a much-vexed one, and we must leave it so. No doubt when statistics are more largely accumulated, showing the relative influence of the various modes of treatment upon (*a*) the life of the patient, (*b*) the duration of the disease, and (*c*) the utility of the resulting impaired joints, more definite rules of practice will be formulated. The mortality of excision is very slight when performed before the existence of a septic sinus. In the after treatment, the object aimed at is to secure a firm union between the remaining portion of the neck of the femur and the acetabulum—not a loose union; the latter would greatly weaken the limb, and would cause greater shortening. During the after treatment, therefore, the limb should be fixed in a Thomas's splint in a strongly abducted position, and with firm bandaging round the pelvis, to keep the hip and femur in close apposition. Those cases of hip disease which are advanced, and where there is extensive bone disease and burrowing of putrid pus, are unsuitable for excision, and must be treated by thorough drainage and cleanliness, or by amputation.

